# Symbiosis with *Francisella tularensis* provides resistance to pathogens in the silkworm

**DOI:** 10.1038/srep31476

**Published:** 2016-08-10

**Authors:** Jin Suzuki, Akihiko Uda, Kenta Watanabe, Takashi Shimizu, Masahisa Watarai

**Affiliations:** 1 The United Graduate School of Veterinary Science, Yamaguchi University, Yamaguchi, Japan; 2 Department of Veterinary Science, National Institute of Infectious Diseases, Tokyo, Japan; 3 Joint Faculty of Veterinary Medicine, Laboratory of Veterinary Public Health, Yamaguchi University, Yamaguchi, Japan

## Abstract

*Francisella tularensis*, the causative agent of tularemia, is a highly virulent facultative intracellular pathogen found in a wide range of animals, including arthropods, and environments. This bacterium has been known for over 100 years, but the lifestyle of *F. tularensis* in natural reservoirs remains largely unknown. Thus, we established a novel natural host model for *F. tularensis* using the silkworm (*Bombyx mori*), which is an insect model for infection by pathogens. *F. tularensis* established a symbiosis with silkworms, and bacteria were observed in the hemolymph. After infection with *F. tularensis*, the induction of melanization and nodulation, which are immune responses to bacterial infection, were inhibited in silkworms. Pre-inoculation of silkworms with *F. tularensis* enhanced the expression of antimicrobial peptides and resistance to infection by pathogenic bacteria. These results suggest that silkworms acquire host resistance via their symbiosis with *F. tularensis*, which may have important fitness benefits in natural reservoirs.

*Francisella tularensis* is a facultative intracellular bacterium and the etiological agent of tularemia, which is highly pathogenic in humans and animals^1^,^2^. Tularemia can be induced by various transmission routes, such as insect bites, inhalation of contaminated aerosols, and ingestion of contaminated food or water^3^. *F. tularensis* has a wide range of animal hosts, such as rabbits, hares, voles, and other rodents^3^, but its life cycle in nature is not fully understood. In addition to mammals, it has also been isolated from environmental water, mud, and mosquito larvae collected in endemic areas^4^,^5^. Therefore, it is thought that *F. tularensis* may persist in natural waters, and the vector-borne transmission of tularemia to mammalian hosts has an important role in the pathogenesis of the disease. In addition, interactions between *F. tularensis* and arthropods play major roles in the ecology of this bacterium and its maintenance in the environment^5^. Deer flies, horse flies, ticks, and mosquitoes are common arthropod vectors for its transmission between mammals^4^, but symbiosis between the bacterium and arthropods is still unclear.

Eukaryotes have evolved and diversified in the context of persistent colonization by non-pathogenic microorganisms^6^. The benefit of symbiosis can be attributed to two types of interaction. The first interaction is symbiosis as a source of novel capabilities, which is based on metabolic or other traits possessed by the microbial partner but not the eukaryotic host^6^. By exploiting these capabilities, eukaryotes have repeatedly derived enhanced nutrition^7^,^8^, defenses against natural enemies^9^,^10^, or other selectively important characteristics^11^. The second interaction is the symbiotic basis of health, which comprises improved vigor and fitness gained by eukaryote hosts via the microbial modulation of multiple traits, including growth rate^12^, immune function^13^,^14^, nutrient allocation^15^, and behavior^16^.

Symbiosis between *F. tularensis* and insects is completely uncharacterized. Thus, here we established a novel symbiosis model for the bacterium in the silkworm *Bombyx mori. B. mori* larvae have been employed as infection models for a large variety of agents, including bacteria, viruses, and fungi^17^,^18^,^19^,^20^. Silkworms can provide a useful model for analyzing innate immunity because insects and mammals share common innate immune mechanisms. Therefore, silkworms provide a useful model for studying host–pathogen interactions in the presence of innate immunity. Compared with mammals, these non-mammalian models have logistical, budgetary, and ethical advantages. Thus, large numbers of larvae can be simply obtained at a low cost to allow large-scale screening, which would not be possible in mammals. Our results demonstrate that silkworms acquire host resistance to pathogenic bacteria via their symbiosis with *F. tularensis*.

## Results

### Silkworm as a novel host model for *F. tularensis* symbiosis

**Figure 1:**
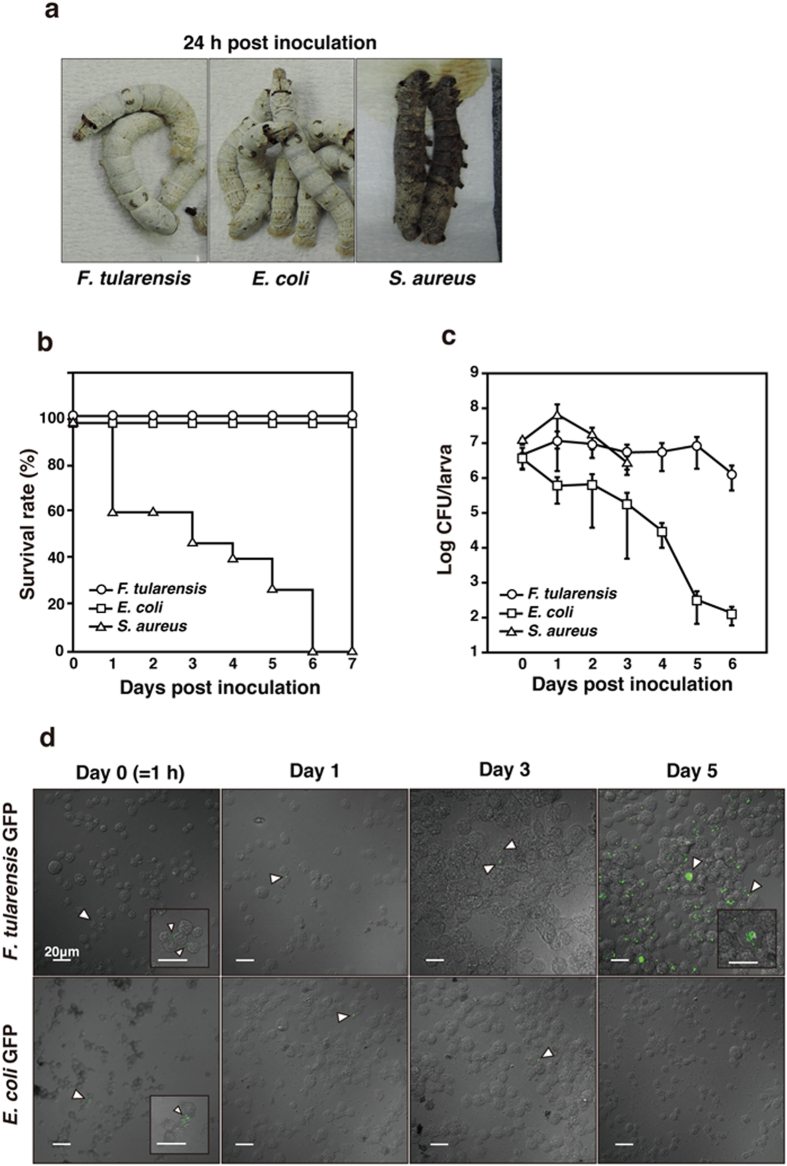
Symbiosis between *F. tularensis* and silkworms. (**a**) Fifth instar larva were infected with *F. tularensis, E. coli*, or *S. aureus.* Larval conditions at 24 h post infection were shown. (**b**) Each silkworm group (n = 15) was infected with *F. tularensis, E. coli* and *S. aureus*, then survival rate was calculated at indicated day point. (**c**) Silkworms were infected with *F. tularensis, E. coli* and *S. aureus*. Bacterial numbers in silkworms were counted at indicated day points. The data represent the averages from triplicate samples based on three identical experiments and the error bars denote the standard error of the mean (n = 9). (**d**) Silkworms were infected with GFP-expressing *F. tularensis* or *E. coli* (arrowheads). Hemolymph cells were collected at indicated day points and observed by confocal laser scanning microscopy. Close-up images are shown in the box frame. Scale bar represents 20 μm.

*F. tularensis* is often found in arthropods such as ticks, but symbiosis between the bacteria and arthropods remains unclear. Thus, we established a novel host model for *F. tularensis* subsp*. holarctica* LVS symbiosis in silkworms. Silkworms were infected with *F. tularensis, Escherichia coli,* or *Staphylococcus aureus* via injection into the hemocoel. Silkworms infected with *F. tularensis* and *E. coli* were alive at 7 days after infection (Fig. 1a,b). No significant differences were observed in the body weight of the silkworms infected with *F. tularensis* and *E. coli* compared with uninfected silkworm (data not shown). By contrast, silkworms infected with *S. aureus* were dead within one day of infection (Fig. 1a,b). We also evaluated the growth of the bacteria in silkworms. We found that the bacterial numbers of *F. tularensis* did not show acute fluctuation for 6 days after infection (Fig. 1c). The bacterial number of *E. coli* decreased each day after infection (Fig. 1c). *S. aureus* exhibited slight growth in silkworms one day after infection but further data could not be obtained because of the decreased growth and death of the silkworms caused by the bacterial infection (Fig. 1c). Green fluorescent protein (GFP)-expressing *F. tularensis* were observed in the hemolymph isolated from silkworms until 5 days after infection (Fig. 1d). By contrast, GFP-expressing *E. coli* were not observed in the hemolymph at 5 days after infection (Fig. 1d). These results suggest that *F. tularensis* establish a symbiosis with the silkworms.

### *F. tularensis* blocks the silkworm immune response trigger

**Figure 2:**
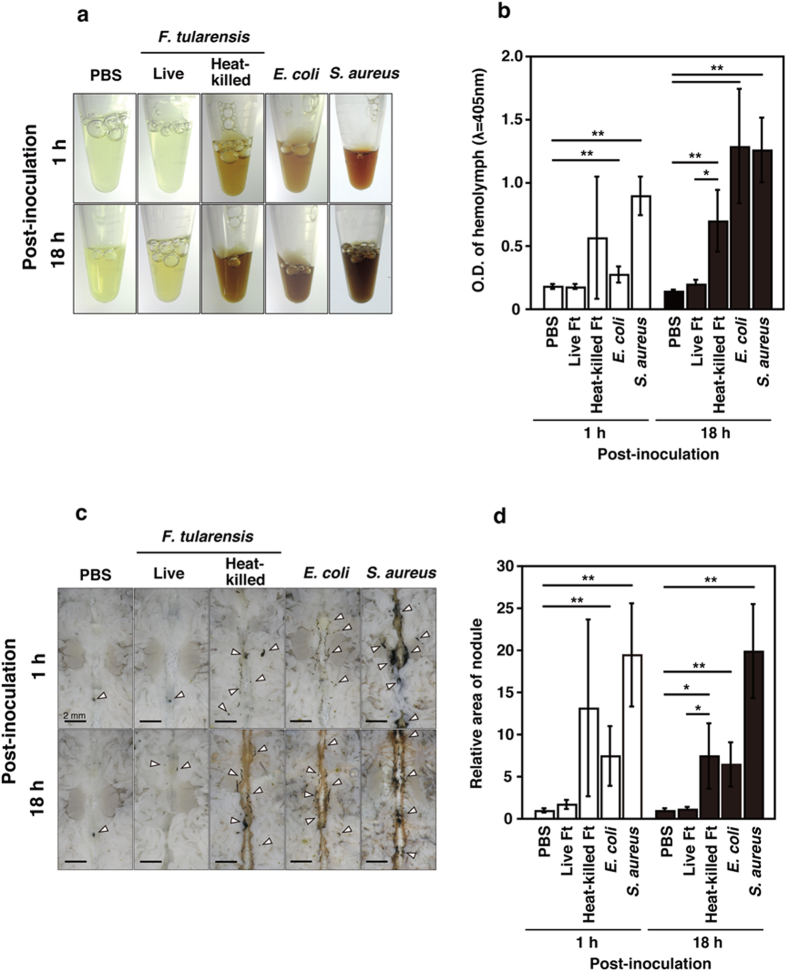
*F. tularensis* inhibits the immune response by silkworms. (**a**) Hemolymph at 1 and 18 h post-inoculation was collected from fifth instar larva inoculated with PBS, live *F. tularensis*, heat-killed *F. tularensis, E. coli*, and *S. aureus*. The condition of melanization was decided by color. (**b**) Silkworms were infected with indicated bacteria, and hemolymph was collected at 1 and 18 h post-inoculation. The optical density (λ = 405 nm) of the hemolymph was measured using a spectrometer immediately after centrifugation to remove hemolymph cells. (**c**) Silkworms were infected with indicated bacteria, and nodule formation around dorsal vessel was observed. Arrowheads indicate nodule formation induced by infected bacteria. (**d**) Silkworms were infected with indicated bacteria, and the total area of nodule formation was calculated using the area measurement tool. The relative melanized area was shown compared to PBS-inoculated control group. (**b**,**d**) The data represent the averages from triplicate samples based on three identical experiments, and the error bars denote the standard deviations. Significant differences were accepted at *P* < 0.05 or *P* < 0.01, and they are indicated by asterisks (*) or double asterisks (**), respectively.

In silkworms, melanization and nodulation are known to be the most common immune responses to bacterial infection^21^. To analyze the melanization and nodulation responses in the silkworms after bacterial infection, we collected hemolymph from the silkworms after bacterial infection and determined the optical density of the samples. Live *F. tularensis* did not induce melanization at 1 and 18 h after infection, whereas heat-killed *F. tularensis* significantly induced melanization at 18 h after inoculation (Fig. 2a,b). *E. coli* and *S. aureus* induced melanization after bacterial infection strongly (Fig. 2a,b). To analyze nodulation, the dorsal vessel was observed by microscopy in silkworms after bacterial infection. *E. coli* and *S. aureus* strongly induced nodulation immediately after bacterial infection (Fig. 2c,d). Live *F. tularensis* did not induce nodulation at 1 and 18 h after infection, whereas heat-killed *F. tularensis* significantly induced nodulation at 18 h after inoculation (Fig. 2c,d). These results suggest that immune responses of silkworm against *F. tularensis* are inhibited during the early stage of the infection, and some activities of *F. tularensis* are involved in the inhibition.

### Silkworm acquires host resistance to *S. aureus* infection after pre-inoculation with *F. tularensis*

**Figure 3:**
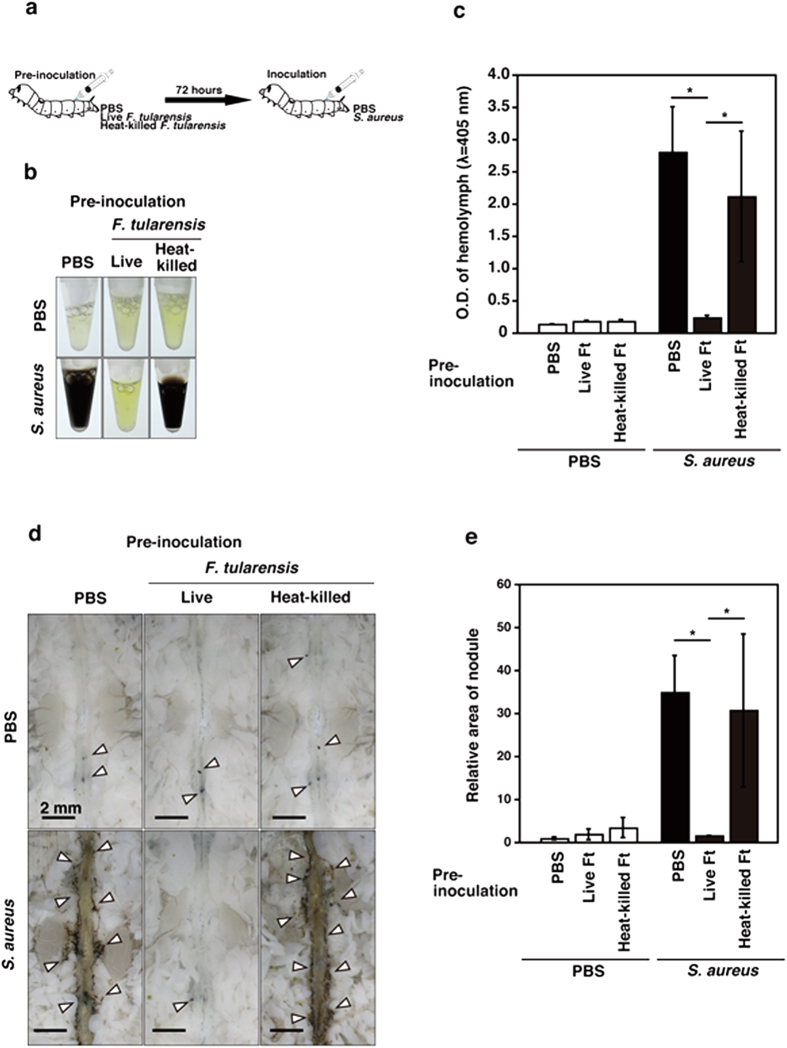
The silkworm immune response induced by *S. aureus* is inhibited by pre-inoculation with *F. tularensis*. (**a**) The experimental scheme is shown. Silkworms were pre-inoculated with indicated bacteria. After 72 h incubation, silkworms were inoculated with PBS or *S. aureus*, and melanization and nodulation were observed. (**b**) Hemolymph was collected from silkworms at 1 h post-inoculation with PBS or *S. aureus*, and the condition of melanization was decided by color. (**c**) The optical density (λ = 405 nm) of the hemolymph collected at 1 h post second inoculation was measured using a spectrometer immediately after centrifugation to remove hemolymph cells. (**d**) Nodule formation around dorsal vessel at 1 h post second inoculation was observed. Arrowheads indicate nodule formation. (**e**) The total area of nodule formation was calculated using the area measurement tool. The relative melanized area of nodule formation was shown compared to PBS-inoculated control group. (**c**,**e**) The data are presented as averages from triplicate samples based on three identical experiments, and the error bars denote the standard deviations. Significant differences were accepted at *P* < 0.05, and they are indicated by asterisks (*).

Melanization and nodulation were not induced by *F. tularensis* infection, so we investigated whether the melanization and nodulation responses induced by *S. aureus* were inhibited by pre-inoculation with *F. tularensis*. Silkworms were inoculated with live or heat-killed *F. tularensis* and incubated for 72 h at room temperature. After 72 h incubation, melanization and nodulation induced by heat-killed *F. tularensis* calmed down. The silkworms were then inoculated with PBS or *S. aureus*, and we measured the melanization and nodulation responses (Fig. 3a). The results showed that pre-inoculation with live *F. tularensis* inhibited the melanization and nodulation responses induced by *S. aureus* infection, whereas the heat-killed bacteria were not effective at immune inhibition (Fig. 3b–e).

**Figure 4:**
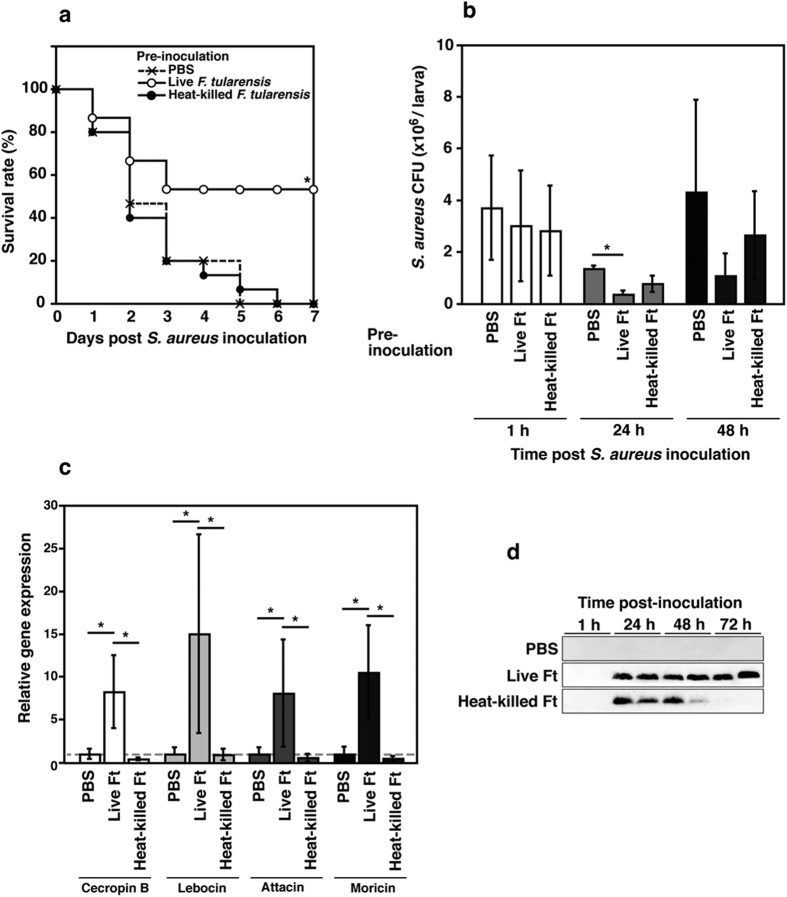
Silkworms acquire host resistance to *S. aureus* infection after pre-inoculation with *F. tularensis.* (**a**) Each silkworm group pre-inoculated with PBS, live *F. tularensis*, or heat-killed *F. tularensis* (n = 15) were inoculated with *S. aureus*, then survival rate was calculated at indicated day point. Significant differences between live and heat-killed *F. tularensis* pre-inoculated groups were accepted at *P* < 0.05 and indicated by asterisk (*). (**b**) Silkworms were pre-inoculated with PBS, live *F. tularensis*, or heat-killed *F. tularensis*. Silkworms were then infected with *S. aureus*. Bacterial numbers of *S. aureus* was counted at 1 h, 24 h, and 48 h post second inoculation. (**c**) RNA samples were collected from the fat bodies of silkworms inoculated with PBS, live *F. tularensis*, or heat-killed *F. tularensis*. The expression levels of AMP genes were determined by real-time PCR. The relative expression levels are presented compared to PBS inoculated group. (**d**) Hemolymph plasma samples from silkworms pre-inoculated with PBS, live *F. tularensis*, or heat-killed *F. tularensis* were collected at 1 h, 24 h, 48 h and 72 h post inoculation. Cecropin B expression levels were analyzed by immunoblotting. (**b**,**c**) The data represent the averages from triplicate samples based on three identical experiments and the error bars denote the standard error of the mean (n = 9). Significant differences were accepted at *P* < 0.05 and they are indicated by asterisks (*).

We hypothesized that pre-inoculation with live *F. tularensis* may have affected the immune response in silkworms, so we investigated the survival rate of silkworms after bacterial infection. We found that silkworms pre-inoculated with live *F. tularensis* survived *S. aureus* infection and they exhibited significant host resistance to bacterial infection compared with those pre-inoculated with heat-killed *F. tularensis* (Fig. 4a). Pre-inoculation with live *F. tularensis* significantly inhibited the growth of *S. aureus* in silkworms at 24 h after infection compared to PBS inoculated control (Fig. 4b). By contrast, heat-killed *F. tularensis* did not affect the survival rate of silkworms or the growth of *S. aureus* in the silkworms (Fig. 4a,b).

Antimicrobial peptides (AMPs) are well-known immune factors that combat pathogens in arthropods^22^. To investigate whether AMPs contribute to the host resistance caused by pre-inoculation with live *F. tularensis*, we analyzed the expression of genes for typical AMPs, i.e., cecropin B, lebocin, attacin, and moricin, at 72 h after inoculation with live or heat-killed *F. tularensis*. We found that the expression levels of these AMP genes were significantly induced by pre-inoculation with live *F. tularensis*, whereas the heat-killed bacteria had no effect (Fig. 4c). We also confirmed the time-course expression of cecropin B by immunoblotting (Fig. 4d). Live *F. tularensis* induced cecropin B expression and the induction was sustained at 72 h post infection. In contrast, the expression induced by heat-killed *F. tularensis* was reduced at 48 h and disappeared at 72 h post infection.

## Discussion

Arthropods are involved in the life cycle of *F. tularensis*^4^. Therefore, the development of arthropod host models is useful for studying the mechanisms related to *F. tularensis* infection and symbiosis. In this study, we established a novel, symbiotic host model for *F. tularensis* using silkworms. The ecology of *F. tularensis* and the natural reservoirs of the bacterium in the environment are not fully understood. The wax moth (*Gallaria mellonella*) has been used as a mammalian infection model for *F. tularensis*^23^, but symbiosis between *F. tularensis* and insects is still unclear. However, we observed a symbiosis between *F. tularensis* and silkworms in the present study; therefore, some types of insect may be candidates as natural reservoirs. Since the silkmoths that hatched from *F. tularensis*-infected larva still retained the *F. tularensis* bacteria (data not shown), it is possible that the animals were infected with *F. tularensis* by eating insects and/or larvae containing the bacteria.

Insects only possess innate immunity^24^; therefore, insects are generally used as models to study the basis of innate immunity. Melanization and nodulation are known to be the first defensive responses to bacterial invaders in arthropods^25^. Melanin can seal off foreign organisms in the hemocoel and starve them of nutrients^26^,^27^. Melanin synthesis also results in the production of reactive oxygen and nitrogen intermediates, which are toxic to some pathogens^28^. Nodule formation is a rapid response that removes microorganisms from the hemocoel. Granulocytes release sticky material after bacterial infection, and the hemocytes and bacterial cells clump together, thereby resulting in the formation of nodules^29^, which comprise aggregations of hemocytes and microorganisms that are subsequently subjected to melanization^30^. By contrast, AMPs might work during a later stage of infection because their production and concentration in the hemolymph both increase after bacterial infection^31^,^32^. In this study, we demonstrated that live *F. tularensis* inhibited melanization and nodulation, but not heat-killed *F. tularensis*, suggesting that some biological activities of *F. tularensis* may inhibit the immune responses. *F. tularensis* posses type VI secretion system which is important for intracellular growth in host cells. This secretion system contribute to control immune system in silkworm. Indeed, the type VI secretion system is reported to be involved in intracellular growth in mosquito cell line^33^. Thus, symbiosis between *F. tularensis* and silkworms may be established by inhibiting the silkworm immune responses during the early stage of infection. The over-activation of immune reactions can damage the host animal itself 34. *S. aureus* has a very rapid growth rate and it causes high mortality in silkworms^20^. However, although *F. tularensis* remained at similar bacterial numbers to *S. aureus* in silkworms, they never died. Thus, the over-activated immune reactions caused by *S. aureus* infection may lead to silkworm death in the early stage of infection.

A key insect-based immunological study discovered Toll in *Drosophila*, which led to the identification of mammalian Toll-like receptors^35^,^36^. Insect Toll functions as a receptor for an endogenous ligand, which relays signals to transcription factors that produce AMPs^37^. The silkworm has 14 Toll isotypes, some of which are expressed several hours after bacterial infection^38^,^39^. These receptors mediate the induced immune responses that are known as pathogen-associated molecular patterns, such as those to lipopolysaccharide and peptidoglycan^40^,^41^. We found that *F. tularensis* inhibited the silkworm immune responses in the early stage of infection, but the production of AMPs was enhanced in the later stage of infection. *F. tularensis* is also sensitive to some AMPs^42^, but the bacterium may escape from the effects of AMPs by endosymbiosis within host cells. Therefore, silkworms engaged in symbiosis with *F. tularensis* exhibited resistance to *S. aureus* infection.

*F. tularensis* can resist degradation in the phagosome and replicate inside mammalian macrophages^43^. We did not demonstrate the phagocytosis activity of hemocytes directly in this study, but intracellular bacteria were clearly observed in the hemocytes at 5 days after infection. Hemocytes may be one of the targets for bacterial invasion and *F. tularensis* may control the immune response in the silkworm to take advantage of suitable conditions for symbiosis. The intracellular signaling pathway related to the expression of AMP in silkworms is still unclear. However, Ishii *et al*. showed that the phosphorylation of p38 MAPK protein conferred protection against *S. aureus* infections in silkworms by up-regulating the expression of AMP genes, but it did not affect the melanization activity^44^. In *Drosophila*, the activation of p38 induces host protection from various bacterial pathogens and fungi because of the up-regulated expression of genes for stress response factors and specific AMPs (cecropin B and attacin)^45^. Thus, *F. tularensis* may induce specific signaling pathways that are affected by intracellular bacteria. Therefore, these signaling pathways may be a possible target for disrupting the lifecycle of *Francisella* in the environment.

Symbiosis is considered to provide benefits to both symbionts. Various insects possess intracellular bacteria within specialized cells known as bacteriocytes, the sole function of which appears to be the housing and maintenance of bacteria^46^. Insect immune effectors have been implicated in the regulation of the bacteria found in the bacteriocytes of the weevil *Sitophilus. Sitophilus* bacteriocytes express a cationic AMP, coleoptericin A, at high levels, and this AMP plays an important role in controlling the maintenance of the symbiont^13^. *Wolbachia pipientis* is an obligate intracellular bacterium and a common endosymbiont of insects^47^. *Drosophila melanogaster* flies infected with *W. pipientis* are less susceptible to the induction of mortality by a range of RNA viruses^48^. *Wolbachia* also inhibits the ability of a range of pathogens, such as *Plasmodium*, dengue virus, and Chikungunya virus, to infect *Aedes aegypti*^49^. The depressed vector competence of *Wolbachia*-infected mosquitoes may be caused by an enhanced immune function, including the induction of AMPs, melanization, and reactive oxygen species^50^. We showed that silkworms in symbiosis with *F. tularensis* were protected from death caused by *S. aureus* infections. Thus, symbiosis with *F. tularensis* may provide fitness benefits for insects, and the human pathogen *F. tularensis* may have an important role in protecting natural reservoirs, such as arthropods, from pathogenic invaders. *F. tularensis* has been isolated from deer flies, horse flies, ticks, and mosquitoes^5^. *Francisella*-like endosymbionts have also been reported in various tick species^51^,^52^,^53^. In this study, our results suggest that *F. tularensis* can infect and survive in endosymbiosis with silkworms; therefore, many other insect species may also be vectors of tularemia. Thus, *Francisella* may be distributed in more arthropod species than considered at present.

## Methods

### Bacterial strains and culture conditions

*Francisella tularensis* subsp*. holarctica* LVS, *Escherichia* c*oli* JM109, *E. coli* JM109 pAcGFP (Clontech, Mountain View, CA), and *Staphylococcus aureus* ATCC923 were used in this study. Bacterial strains were maintained as frozen glycerol stocks. *F. tularensis* subsp*. holarctica* LVS was obtained from the Pathogenic Microorganism Genetic Resource Stock Center, Gifu University and was cultured aerobically at 37 °C in brain heart infusion broth (BD, Franklin Lakes, NJ) supplemented with cysteine (BHIc)^54^ or Brucella broth (BD) containing 1.5% agar (Wako, Osaka, Japan). *E. coli* JM109, and *S. aureus* ATCC923 were cultured in Luria–Bertani (LB) broth (Nacalai Tesque, Kyoto, Japan) or LB broth containing 1.5% agar. Ampicillin (100 μg/mL) and chloramphenicol (10 μg/mL) were used as necessary.

### Establishment of a GFP-expressing *F. tularensis* strain

A GFP-expressing plasmid, pNVU-GFP, was constructed from a pNVU1-expressing plasmid^55^. The tetracycline resistance gene was removed from pNVU1 by treating the plasmid with *Sma*I. The GFP gene containing an SD sequence was amplified from pGreenTIR^56^ using the primer pairs pNVU-GFP-F (5′-GAAATTATTGATCCCTGATTAACTTTATAAGGAGGAA-3′) and–R (5′-CTTGGTCTGACACCCCTATTTGTATAGTTCATCCATG-3′), and inserted into *Sma*I-digested pNVU1. pNVU-GFP was transformed and replicated in *E. coli* DH5α and purified using a Plasmid Midi Kit (Qiagen, Hilden, Germany). LVS was transformed with pNVU-GFP by electroporation. The transformed LVS was cultured in BHIc for 3 h and then selected on BHIc agar plates containing 5 μg/mL chloramphenicol.

### Silkworms

Fourth instar *B. mori* larvae (Hu/Yo × Tukuba/Ne) were obtained from Ehime-Sanshu (Ehime, Japan). The larvae were raised by feeding them with Silkmate 2M (Nosan Corporation, Kanagawa, Japan) at room temperature (25 °C).

### Infection using silkworm larva

Day 2 fifth instar larva was inoculated in the hemocoel with 50 μL of bacterial solution containing 1 × 10^8^ CFU/mL in PBS using a 1-mL syringe equipped with a 30-gauge needle (Terumo Inc., Tokyo, Japan). After inoculation, the silkworms were incubated at room temperature with food. To obtain bacterial counts (as CFU/mL), the infected silkworm larvae were weighed and placed in disposable 15-mL centrifuge tubes, before homogenizing with a Biomasher SP (Funakoshi Co., Ltd, Tokyo, Japan) and suspending in 3 mL of PBS. The suspension was subsequently centrifuged at 300 × *g* for 30 s and solid tissues were separated from the concentrated suspension. Using appropriate dilutions, the suspension samples were spread onto agar plates and the numbers of colonies were counted. To calculate the counts (CFUs), the summed volumes of the hemolymph and tissues were estimated together (1 g = 1 mL).

### *In vivo* melanization analysis

Day 2 fifth instar larva was inoculated with 50 μL of bacterial solution containing 2 × 10^8^ CFU/mL in PBS. Control groups were injected with PBS or an equal volume of 75 °C/30-min heat-killed *F. tularensis*. After 1 h and 18 h, the hemolymph was collected from the caudal horn and placed in a pre-chilled 1.5-mL tube on ice to prevent further melanization because of exposure to the air. The hemolymph samples were centrifuged at 6000 × *g* for 5 min at 4 °C to remove the hemolymph cells. The optical density (λ = 405 nm) of each supernatant fraction was measured using a spectrometer immediately after centrifugation.

### *In vivo* nodule formation analysis

Melanized nodules precipitated around the dorsal vessel were observed, as described previously^29^. Photographs were taken from the seventh to ninth segments under the same conditions using a stereoscopic microscope. To quantify the formation of nodules, each photograph was imported into Image J 1.42l software (NIH, USA; http://rsbweb.nih.gov/ij/), and the total amount of pixels in each sample was calculated with the ImageJ area measurement tool. The relative melanized area was calculated as the ratio of each group relative to that of the control.

### Fluorescence microscopy

GFP-expressing bacteria were used to inoculate fifth instar day 2 larvae, which were then incubated at room temperature with food. At 1, 24, 72, and 120 h post-inoculation, hemolymph was collected from the caudal horn and added to a 24-well tissue culture plate, before diluting up to 500 μL with IPL-41 Insect Medium (Sigma-Aldrich, St Louis, MO) supplemented with 10% heat-inactivated fetal bovine serum. The plates were then centrifuged for 5 min at 900 × *g* and incubated for 15 min at room temperature. After washing twice with PBS, the samples were fixed with 4% paraformaldehyde (Wako, Osaka, Japan) in PBS for 15 min at room temperature. Subsequently, the samples were washed twice with PBS. Fluorescent images were obtained using a FluoView FV100 confocal laser scanning microscope (Olympus).

### Pre-inoculation of silkworms

We injected 50 μL of PBS, 1 × 10^8^ CFU/mL live *F. tularensis*, or the same amount of 75 °C/30-min heat-killed *F. tularensis* into each silkworm during pre-inoculation (Fig. 3a). After incubation for 72 h at room temperature with food, the silkworms were inoculated with 50 μL of PBS suspension containing 2 × 10^8^ or 1 × 10^7^ CFU/mL *S. aureus* to obtain the survival curve or internal CFU measurements, respectively. For the *in vivo* melanization and nodule formation analyses, each silkworm was injected with 50 μL of 1 × 10^8^ CFU/mL *S. aureus* suspension or PBS as a control to determine the effects of pre-inoculation.

### RNA isolation and qPCR analysis of AMPs

To analyze the expression of AMP genes, we collected the fat bodies from silkworms dissected at 72 h after pre-inoculation. The total RNA was isolated from the fat body using NucleoSpin RNA (Macherey-Nagel, Düren, Germany). The RNA was quantified by absorption at 260 nm using a NanoDrop 2000 (Thermo Fisher Scientific Inc., MA). Reverse transcription was conducted using ReverTra Ace qPCR RT Master Mix (Toyobo Co. Ltd, Osaka, Japan), and cDNA samples were stored at −30 °C prior to use. Next, qPCR was performed with the StepOne Real-Time PCR system (Applied Biosystems, CA, USA) using KOD SYBR qPCR Mix (Toyobo). The primer sets were described previously^57^. The *Actin A3* amplicon was used as an internal control to normalize all of the data. The relative expression levels of the AMPs were calculated using the relative quantification method (∆∆Ct).

### Immunoblotting

Day 2 fifth instar larva was inoculated with 50 μL of PBS, live or 75 °C/30-min heat-killed *F. tularensis* solution containing 2 × 10^8^ CFU/mL in PBS. After 1 h, 24 h, 48 h and 72 h, the hemolymph was collected from the caudal horn and centrifuge (6000 × *g*, 4 °C) for 10 min to isolate hemolymph plasma. The proteins in 1.5 μL of hemolymph plasma were separated by SDS-PAGE with 4–12% Bis-Tris Gel (Thermo Fisher Scientific Inc.), and transferred onto polyvinylidene difluoride membranes (Millipore, Billerica, MA). After blocking with 5% nonfat dry milk in Tris-buffered saline (TBS) at room temperature for 2 h, the membranes were incubated overnight with anti-cecropin B antibody (1:1000; ab27571; Abcam plc, Cambridge, UK) at 4 °C. After washing with TBS containing 0.02% (v/v) Tween 20, the membranes were incubated for 1 h with horseradish peroxidase-conjugated secondary antibody (0.01 μg/mL) at room temperature and immunoreactions were visualized using the enhanced chemiluminescence detection system (GE Healthcare Life Science, Little Chalfont, UK).

### Statistical analysis

Statistical analyses were performed using one-way ANOVA with the post hoc Tukey–Kramer test. Statistically significant differences between groups were accepted at *P* < 0.05 or *P* < 0.01. The survival curves were estimated with the Kaplan-Meier method and the log-rank test was used to determine significant differences between the live and heat-killed *F. tularensis* pre-inoculated groups (*P* < 0.05).

## Additional Information

**How to cite this article**: Suzuki, J. *et al*. Symbiosis with *Francisella tularensis* provides resistance to pathogens in the silkworm. *Sci. Rep.* **6**, 31476; doi: 10.1038/srep31476 (2016).
